# Genome and Evolutionary Analysis of *Nosema ceranae*: A Microsporidian Parasite of Honey Bees

**DOI:** 10.3389/fmicb.2021.645353

**Published:** 2021-06-02

**Authors:** Qiang Huang, Zhi Hao Wu, Wen Feng Li, Rui Guo, Jin Shan Xu, Xiao Qun Dang, Zheng Gang Ma, Yan Ping Chen, Jay D. Evans

**Affiliations:** ^1^Honeybee Research Institute, Jiangxi Agricultural University, Nanchang, China; ^2^Jiangxi Province Key Laboratory of Honeybee Biology and Beekeeping, Jiangxi Agricultural University, Nanchang, China; ^3^Guangdong Key Laboratory of Animal Conservation and Resource Utilization, Guangdong Public Laboratory of Wild Animal Conservation and Utilization, Institute of Zoology, Guangdong Academy of Sciences, Guangzhou, China; ^4^College of Animal Sciences (College of Bee Science), Fujian Agriculture and Forestry University, Fuzhou, China; ^5^College of Life Sciences, Chongqing Normal University, Chongqing, China; ^6^US Department of Agriculture-Aricultural Research Service (USDA-ARS) Bee Research Laboratory, Beltsville, MD, United States

**Keywords:** honey bee, parasite, microsporidia, phylogeny, transporter, long reads

## Abstract

Microsporidia comprise a phylum of single cell, intracellular parasites and represent the earliest diverging branch in the fungal kingdom. The microsporidian parasite *Nosema ceranae* primarily infects honey bee gut epithelial cells, leading to impaired memory, suppressed host immune responses and colony collapse under certain circumstances. As the genome of *N. ceranae* is challenging to assembly due to very high genetic diversity and repetitive region, the genome was re-sequenced using long reads. We present a robust 8.8 Mbp genome assembly of 2,280 protein coding genes, including a high number of genes involved in transporting nutrients and energy, as well as drug resistance when compared with sister species *Nosema apis*. We also describe the loss of the critical protein *Dicer* in approximately half of the microsporidian species, giving new insights into the availability of RNA interference pathway in this group. Our results provided new insights into the pathogenesis of *N. ceranae* and a blueprint for treatment strategies that target this parasite without harming honey bees. The unique infectious apparatus polar filament and transportation pathway members can help to identify treatments to control this parasite.

## Introduction

As the earliest branch from the fungal kingdom, microsporidia comprise a large and widespread group of obligate intracellular animal parasites (Keeling and Fast, 2002; Williams, 2009; Capella-Gutiérrez et al., 2012). In humans, microsporidia are opportunistic parasites that infect immuno-compromised patients (Didier, 2005). Microsporidia also showed substantial damage to the silkworm and fisheries industries and are a driving factor for honey bee colony losses which seriously threaten the agricultural economy and global food security (Higes et al., 2008; Aizen et al., 2009; Freeman and Sommerville, 2011; Stentiford et al., 2016; Santhoshkumar et al., 2017; Meng et al., 2018). In response to their intracellular parasitic life cycle, microsporidia have undergone massive reductions in gene content, including decayed glycolytic pathways, leading to extremely compact eukaryotic genomes (Pelin et al., 2015, 2016; Ndikumana et al., 2017; Wiredu Boakye et al., 2017). Strikingly, mitochondrial genes were lost completely, leaving only a mitochondrion-related organelle called the mitosome (Burri et al., 2006). As a result, energy and resources needed for the proliferation of the parasite are acquired directly from the host, causing energetic stress (Mayack and Naug, 2009; Martin-Hernandez et al., 2011).

*Nosema ceranae* is a microsporidian parasite which infects honey bee mid-gut epithelial cells (Fries et al., 1996). As with all microsporidian parasites, *N. ceranae* produce infectious spores. The spore wall, which is comprised of an electron-dense proteinaceous exospore and an electron-transparent endospore protects the parasite from environmental stressors, allowing spores to remain infective for long term (Li et al., 2003; Fayer, 2004). *N. ceranae* infection starts from the ingestion of spores contaminated nectar and transmitted through oral-fecal and oral-oral routes. The spores germinate in the gut lumen and extrude a polar tube which ejects the sporoplasm into the host cytoplasm (Klee et al., 2007). This leads to parasite proliferation in the subsequent 4 days, resulting in a huge number of offspring spores. The life cycle destroys the gut membrane matrix and epithelial cell integrity (Higes et al., 2006, 2007). Infected honey bees showed suppressed immune responses, impaired memory, and energetic stress (Antunez et al., 2009; Mayack and Naug, 2009; Higes et al., 2013; Gage et al., 2018).

This paper aims to improve the *N. ceranae* genome assembly using long-read sequencing technology, reducing redundancies and improving the integrity of the assembled genome. We also more fully analyzed the transporters, spore wall, and polar tube proteins, essential proteins that fuel proliferation and determine the success of infections. These analyses improve our understanding of parasite evolution but also provide targets to treat an important bee disease.

## Materials and Methods

### DNA Sequencing and Genome Assembly

*N. ceranae* spores were collected from the midgut tissues of heavily infected honey bee colonies. As the genetic diversity of *N. ceranae* is higher within a colony than among colonies, the impacts of multi-colony spores on the quality of the assembled genome was minor (Gómez-Moracho et al., 2014, 2015). The spores were purified using a Percoll gradient procedure and genomic DNA was extracted using the CTAB protocol (Chen Y. et al., 2013). The species status of *N. ceranae* was confirmed by species-specific PCR (Fries et al., 2013). A library was prepared and sequenced following the Oxford Nanopore protocol using MinION cell. Long reads were self-corrected and assembled using Mecat (version 1.0) with default parameters (Xiao et al., 2017). Redundant contigs were collapsed using redundans (version 0.13c) with default parameters (Pryszcz and Gabaldón, 2016). The assembly was aligned against the NCBI bacteria database and honey bee genome by BLASTN to remove contamination. The long reads were re-aligned to the assembled genome to determine structural variations (SVs) using the Ngmlr and Sniffles pipline (Sedlazeck et al., 2018). The assembled genome has been deposited in GenBank with assembly accession number GCA_004919615.1. The raw reads were deposited to NCBI BioProject PRJNA514060.

### Gene Prediction and Functional Annotation

Previously, we quantified *N. ceranae* gene expression profiles at various proliferation stages with RNA-seq (Huang et al., 2019). To improve the gene annotation, we re-mapped those reads to the current *N. ceranae* genome assembly and retrieved the aligned reads using Hisat2 with default parameters (Kim et al., 2013). Both the assembled contigs (Supplementary File 1) and RNA-seq reads were imported into GenSAS, a free online gene features annotation pipeline (Humann et al., 2019). Briefly, the genomes were first masked using RepeatMasker and RepeatMolder (Smit et al., 2015a,b). Next, the genes were predicted using GeneMarkES and Augustus (Lomsadze et al., 2005; Stanke et al., 2008). After that, RNA-seq reads were assembled to longer transcripts using Spades, and these reads were used to polish the annotated gene features using PASA (Haas et al., 2008; Bankevich et al., 2012). In order to infer biological function, the predicted protein sequences were aligned by BLASTN to the Pfam, Uniprot, and NCBI non-redundant databases. The completeness of the assembly was gauged using BUSCO (version 4) against the microsporidia_odb10 dataset (Simao et al., 2015; Seppey et al., 2019).

### Phylogenetic Analysis of the Microsporidian Species

The protein sequences of other 19 microsporidian parasites (*Encephalitozoon romaleae*, *Encephalitozoon hellem*, *Encephalitozoon intestinalis*, *Encephalitozoon cuniculi*, *Ordospora colligata*, *Nosema apis*, *Nosema bombycis*, *Enterocytozoon bieneusi*, *Enterospora canceri*, *Enterocytozoon hepatopenae*, *Vittaforma corneae*, *Trachipleistophora hominis*, *Vavraia culicis*, *Pseudoloma neurophilia*, *Edhazardia aedis*, *Anncaliia algerae*, and *Nematocida parisii*) with assembled genomes were retrieved from NCBI and MicrosporidianDB^1^,^2^ (Katinka et al., 2001; Corradi et al., 2007, 2009, 2010; Cuomo et al., 2012; Heinz et al., 2012; Pombert et al., 2012, 2013, 2015; Campbell et al., 2013; Chen Y. et al., 2013; Pan et al., 2013; Haag et al., 2014; Desjardins et al., 2015; Ndikumana et al., 2017; Reinke et al., 2017; Wiredu Boakye et al., 2017). These protein sequences were all used to query the BUSCO gene set microsporidian_odb 10 (Simao et al., 2015; Seppey et al., 2019). The 48 shared BUSCO groups among all 20 microsporidian species were aligned using Muscle with default parameters (Edgar, 2004). Resulting alignments were trimmed with trimAI (-w 3 -gt 0.95 -st 0.01) and then concatenated for phylogenetic analyses with Mrbayes (nchain = 4, aamodelpr = mixed, ngen = 1,000,000) (Ronquist and Huelsenbeck, 2003). The tree was then viewed and edited using FigTree^3^. The species *M. daphniae* was used to root the tree.

### Synteny and Phylogenic Analysis of the Gene *Dicer*

A paired synteny analysis among *N. ceranae*, *N. apis*, and *N. bombycis* was performed and viewed using SyMAP (V5.0.5) (Soderlund et al., 2011). In order to further analyze the selection of the gene *Dicer*, protein sequences were retrieved from 11 microsporidian species that have maintained the gene encoding *Dicer*. These *Dicer* orthologs have been described previously (Ndikumana et al., 2017). The sequences were aligned with Muscle, and a phylogenic tree was constructed using Mrbayes (nchain = 4, aamodelpr = mixed, ngen = 1,000,000) (Ronquist and Huelsenbeck, 2003; Edgar, 2004). Additionally, as the intergenic regions were fragmented, the maximal length of nucleotides up-(1 Kbp) and down-(2 Kbp) stream of the gene for *Dicer* were retrieved and aligned with Muscle (Edgar, 2004). These aligned sequences were then concatenated for the phylogenetic calculation with Mrbayes (nchain = 4, rates = invgamma, ngen = 1,000,000) (Ronquist and Huelsenbeck, 2003). The species *M. daphniae*, *A. algerae*, and *N. apis* were excluded, as the gene for *Dicer* was located at either the start or end of contigs or at a gap, where the nucleotides up- or down-stream of the gene for *Dicer* were not long enough to perform phylogenic analysis.

### Synteny Analysis of Polar Tube Proteins (PTP) and the Identification of Spore Wall Proteins (SWP)

In order to further characterize the PTP genes and SWP genes, protein and CDS sequences encoded SWP were downloaded from NCBI and used as a library. The genome sequences of *N. ceranae* were used to query this library. Through blast with an E-value cutoff of ≤1e−5, the best aligned sequence was used for further analysis. To identify PTPs in *N. ceranae*, about 10 kb sequences of upstream and downstream of PTPs in *E. cuniculi* and *N. bombycis* were obtained, then candidate sequences were retrieved by BLAST (*E*-value ≤ 1e−10). Synteny blocks were identified manually from BLAST coordinates.

### Identification and Phylogenetic Analyses of Iron-Sulfur (Fe-S) Cluster Assembly Machinery

The components of the Fe-S cluster assembly machinery have been preliminarily identified for the microsporidians *E. cuniculi* and *T. hominis* (Goldberg et al., 2008). To identify the Fe-S cluster assembly proteins for *N. ceranae*, the amino acid sequences of *E. cuniculi* and *T. hominis* Fe-S cluster assembly genes *Isu1*, *Nfs1*, and *Hsp70* gene were aligned to *N. ceranae* protein set using BLASTP. Putative Fe-S cluster assembly genes for *N. ceranae* were designated by E-value ≤ 1e−20 and query coverage ≥ 95%, and only one record for each gene was obtained. Sequences were aligned using Muscle (Edgar, 2004). The phylogenetic trees were built up using the PhyML program with the WAG model under maximum likelihood (Anisimova and Gascuel, 2006). The TreeDyn program was applied to visualize the trees (Chevenet et al., 2006). All the above analyses from the sequence alignment to tree reconstruction were carried out on the phylogeny.fr platform (Dereeper et al., 2008). Outputs in Newick format from this platform were downloaded and further used as input in the iTOL program to generate an unrooted, circular phylogenetic tree (Letunic and Bork, 2007).

### Identification and Phylogenetic Analyses of the ATP Binding Cassettes (ABC) Transporter and ATP/ADP Carriers

The protein sequences of 22 fungal species were downloaded from NCBI, including 20 microsporidian species (*E. romaleae*, *E. hellem*, *E. intestinalis*, *E. cuniculi*, *O. colligata, N. apis, N. bombycis, E. bieneusi, E. canceri, Enterocytozoon hepatopenae, V. corneae, T. hominis, V. culicis, P. neurophilia, E. aedis, A. algerae, Nematocida* spERTm5, *Nematocida* sp1, *N. parisii, M. daphniae)* and two yeast species (*Schizosaccharomyces pombe* and *Saccharomyces cerevisiae*). The sequences of fungal ABC transporters were obtained from previous studies, which were used as seed sequences to query the downloaded protein sequence sets using BLAST with cutoff *P* < 0.05 (Paumi et al., 2009; Kovalchuk and Driessen, 2010). Sequences were examined manually to remove apparently incomplete sequences against query seeds. All candidate ABC amino acids were then aligned with MAFFT (Katoh et al., 2005). Amino acid substitution models for the ABC family were selected based on Prottest3 (Darriba et al., 2011). The phylogenetic tree was generated using FastTree based on the Jones-Taylor-Thornton (JTT) model with 1,000 bootstraps (Price et al., 2009). The final tree was viewed with MEGA7 (Kumar et al., 2016).

Additionally, protein sequences of ATP/ADP carriers for *S. cerevisiae, E. cuniculi* and *E. bieneusi* were downloaded from NCBI and then used to query against the microsporidian protein sequences using BLAST with *P* < 0.05 as cutoff. Sequences were examined manually to remove apparently incomplete sequences against query seeds. All candidate protein sequences were then aligned with MAFFT (Katoh et al., 2005). Amino acid substitution models for ATP/ADP carriers were selected based on Prottest3 (Darriba et al., 2011). A phylogenetic tree was generated using PhyML based on the Jones-Taylor-Thornton (JTT) model with 1,000 bootstraps (Guindon and Gascuel, 2003). The final tree was viewed with MEGA7 (Kumar et al., 2016).

## Results and Discussion

### Genome Assembly Statistics, Gene Features and Completeness

**FIGURE 1:**
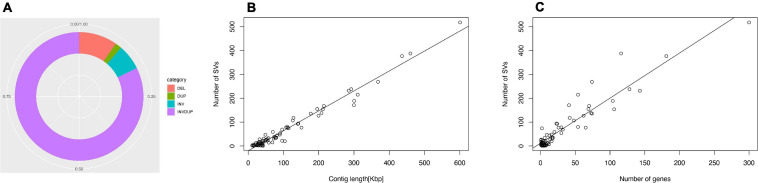
Structural variation of the *N. ceranae* genome. **(A)** In total, 8209 SV events were identified. The inversion duplication dominated the SV events, followed by indels, inversion and duplication. **(B)** The number of SV events is positively correlated with the contig length. **(C)** The number of SVs within genes.

**FIGURE 2:**
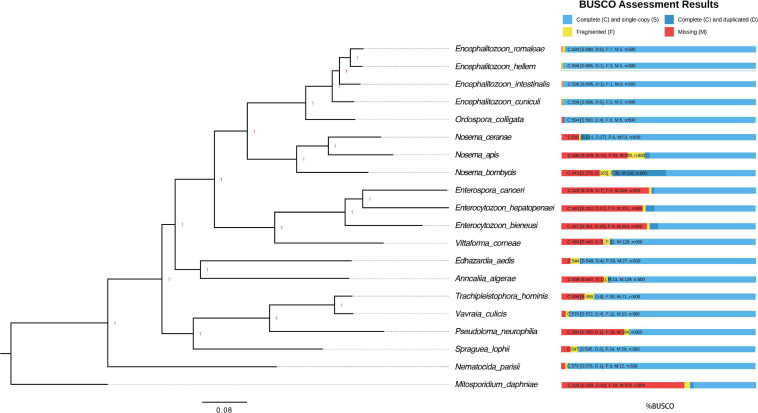
Phylogenetic tree and estimated completeness of the genome of 20 microsporidian species. The phylogenic tree was constructed based on 48 BUSCO genes shared among the microsporidian species and *M. daphniae* is used to root the tree. All the branches were 100% supported. Overall, 90.2% of microsporidian BUSCOs (V10) were identified from *N. ceranae*. The studied species N. ceranae has been highlighted. The RNAi genes have been lost twice without reversal, which have been highlighted.

The genomes of microsporidian parasites are generally compact due to their intracellular parasitic life history, ranging from 2–15 Mbp (Ndikumana et al., 2017). However, an exceptionally large genome of 51 Mpb has also been observed (Desjardins et al., 2015). Within the microsporidian parasites, the species maintained RNAi genes also showed a number of transposable elements, which might contribute to the observed larger genome sizes (Ndikumana et al., 2017). The genome of *N. ceranae* is notoriously difficult to assemble due to an extremely high level of within-colony genetic diversity (Gómez-Moracho et al., 2014) and the inability to produce pure cultures outside of honey bee hosts. In our study, 2,186,226 reads were generated, resulting in an 8.8 Mbp assembled genome (1,141× genome coverage). The genome was composed of 110 contigs, a substantial improvement over the previous assembly. In total, 2,280 genes were predicted with an average length of 1,057 nucleotides per gene, all supported by transcriptomic reads (Table 1 and Supplementary File 1). Alternative splicing has not been found, but 3′ UTR were identified, which enhanced miRNA-targeted gene prediction. By aligning the long reads back to the assembly, 97.2% of reads can be aligned. The majority of structural variations (SVs) were inversion duplications, and the number of SVs were positively correlated with contig length (Figure 1). The impacts of SVs on any phenotypic effects in microsporidia remain unclear, even though SVs contribute to genomic diversity (Borneman et al., 2011). In the budding yeast, SVs were suggested to be involved in tolerance toward stressors (Zhang et al., 2016). Also, SVs in fission yeasts showed strong impacts on quantitative traits and reproductive isolation (Jeffares et al., 2017). In our study, 1,785 genes were found within SVs. By aligning the protein sequences to the KEGG database, the distribution of genes among the six functional categories was not significantly different across the genome (Pearson’s Chi-squared test, *P* > 0.05). By aligning the RNA-seq reads back to the assembly, all the predicted protein-coding genes were expressed, suggesting these protein-coding genes were functional. Out of 600 conserved BUSCO groups, 541 complete, 6 fragmented and 53 missing BUSCOs were identified, indicating the assembly is nearly complete (Figure 2). As the BUSCO gene sets for microsporidia were primarily based on *Encephalitozoon* species, genes could be lost during the divergence, such as RNAi genes, which might partly explain the missing BUSCO genes in microsporidian species. Overall, the genome-based phylogenetic tree is consistent with previous ones (Ndikumana et al., 2017).

**TABLE 1:** Assembly statistics of three versions of *N. ceranae* genome.

Assembly statistics	GCA_004919615.1	GCA_000988165.1	GCA_000182985.1
Sequencing technology	Oxford Nanopore	Illumina HiSeq	454
Genome coverage	1,141	120	25
Assembly size (Mbp)	8.8	5.6	7.8
Number of contigs	110	536	5,465
N50 (Kbp)	177.3	42.5	2.9
Number of protein-coding genes	2,280	3,246	2,060
Percentage of genes supported by RNA-seq	100%	93%	95%
Number of aligned BUSCO	541	583	508

### Synteny and Phylogenetic Analysis of *Dicer*

**FIGURE 3:**
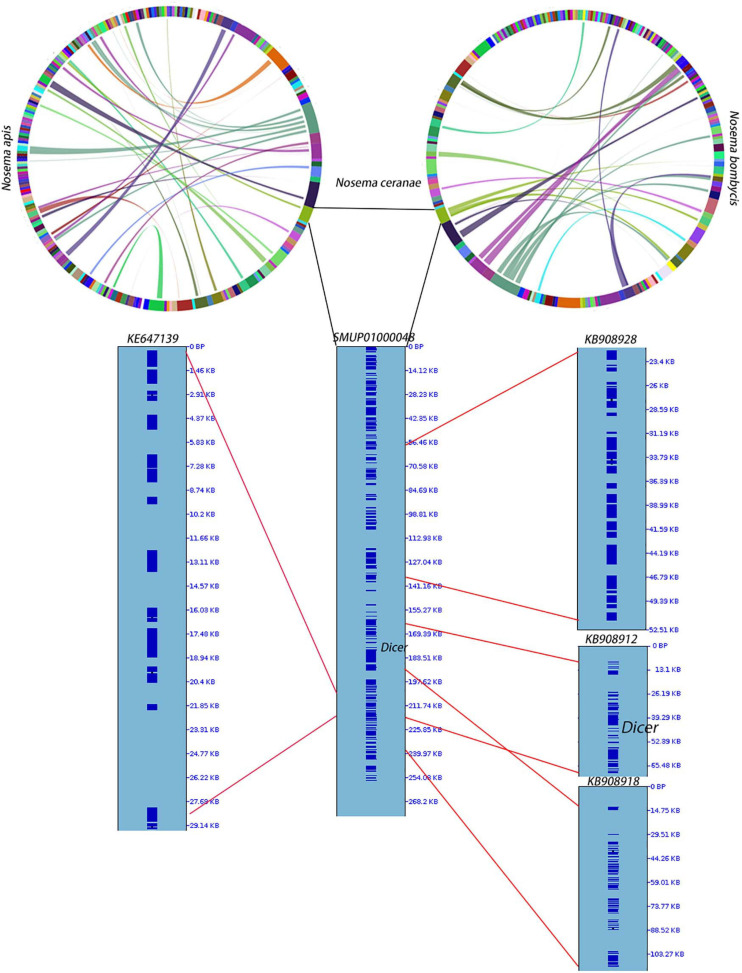
Synteny among *N. apis*, *N. ceranae* and *N. bombycis*. At the genome level, 28 synteny blocks were identified between *N. bombycis* and *N. ceranae*. Additionally, 34 synteny blocks were identified between *N. apis* and *N. ceranae*. Surprisingly, a synteny block with the gene *Dicer* is not shared among the three species. The gene *Dicer* was predicted in *N. ceranae* contig (SMUP01000048). The paired synteny block between *N. ceranae* and *N. apis*, as well as between *N. ceranae* and *N. bombycis* were further shown in this contig. The corresponding region are shown on the right of the contig and the region with red lines indicates a synteny block. A synteny block with the gene *Dicer* was identified between *N. ceranae* and *N. bombycis*.

**FIGURE 4:**
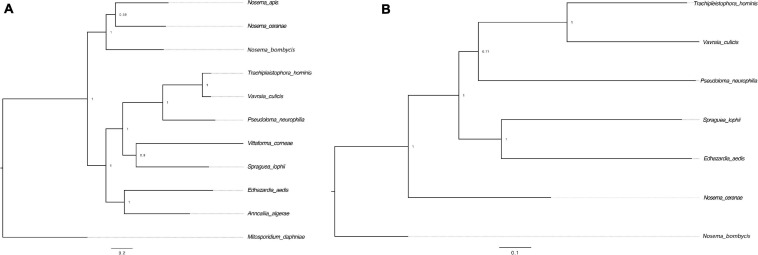
Phylogenetic trees of the gene *Dicer* and surrounding regions. **(A)** The tree was constructed based on the protein sequences of the gene *Dicer*. *M. daphniae* was used to root the tree. **(B)** The tree was constructed based on the nucleotides up and down stream of the gene *Dicer*. *N. bombycis* was selected to root the tree. The congruence of the two trees suggests a hitchhiking effect of *Dicer* during microsporidian divergence.

RNAi is a mechanism to regulate gene expression at the post-transcriptional level, which is crucial for the development and defenses of organisms (Zhao and Srivastava, 2007; Obbard et al., 2009). Based on the assembled genomes, a few microsporidian species have lost RNAi genes (Ndikumana et al., 2017). However, the evolutionary forces driving the loss of RNAi genes in microsporidian species remain unclear. Out of 20 selected microsporidian species, a subset of 11 species has maintained RNAi genes *Dicer* and *Argonaute*, including *M. daphniae*, *S. lophii*, *P. neurophilia*, *V. culicis*, *T. hominis*, *A. algerae*, *E. aedis*, *V. corneae*, *N. bombycis*, *N. apis*, and *N. ceranae*. We did not find evidence suggesting that the loss of RNAi genes was associated with host specificity, either between insect and non-insect, or between vertebrate and invertebrate (Huang, 2018). As the flanking sequences and *Dicer* were selected as a unit, the topology of the phylogenic tree suggests that the loss of RNAi occurred late in the divergence of the microsporidian species twice without reversal. The events of RNAi maintenance significantly deviated from random (Fisher’s Exact test, *P* < 0.05; Figure 2). As *N. ceranae*, *N. apis* and *N. bombycis* were the most closely related sister species, and all three species maintained the gene *Dicer*, a synteny block with the gene *Dicer* is expected among the three species. By pair-wise analyses, 28 synteny blocks were identified between *N. bombycis* and *N. ceranae*, including a synteny block containing the gene *Dicer* (Figure 3). Additionally, 34 synteny blocks were identified between *N. apis* and *N. ceranae*. However, a synteny block containing the gene *Dicer* was not found between these two species, which might be due to a genome rearrangement. Alternatively, the orthologous anchor genes might have been fragmented during the assembly processes of other species (Liu et al., 2018). Therefore, two additional phylogenic analyses were further performed using protein sequences of the gene *Dicer*, as well as the up and downstream nucleotides of the gene *Dicer*. The topology of the phylogenetic tree for *Dicer* was consistent with the proposed divergence of microsporidian species (Figure 4). It is then reasonable to expect signals of genetic hitchhiking around the gene *Dicer* (Barton, 2000). Therefore, 1 Kbp up-stream and 2 Kbp down-stream the gene *Dicer* were extracted to construct a phylogenic tree. The topologies of the trees of both datasets were highly congruent, suggesting the upstream and downstream of the gene *Dicer* were under selection in parallel with the gene *Dicer* without insertion or recombination, at least within the studied clades due to hitchhiking.

### Phylogenetic Analysis of Iron-Sulfur (Fe-S) Cluster Assembly Machinery

Microsporidia are highly obligate intracellular parasites of animals with extremely compact genomes and decreased cellular and biochemical reactions (Katinka et al., 2001; Keeling and Fast, 2002; Corradi et al., 2010). The microsporidian phylum lacks typical mitochondria but contains a mitosome, a tiny mitochondrial remnant (Williams et al., 2002). Although mitosomes have lost canonical mitochondrial functions like aerobic respiration and biosynthesis, their ability to generate Fe-S proteins essential for the maturation of proteins of diverse functions is maintained (Goldberg et al., 2008). Several components are required for the Fe-S cluster assembly machinery of mitosomes. First, the scaffold proteins Isu1 and Isu2 are involved in the *de novo* biosynthesis of a transiently bound Fe-S cluster. In this step, iron and sulfur are donated by frataxin (Yfh1) and the cysteine desulphurase complex Nfs1–Isd11, respectively. The electrons are delivered by ferredoxin (Yah1). Then, the Fe–S cluster pass from Isu1 and Isu2 to target apoproteins with the support from a Hsp70 (Ssq1) protein, co-chaperone Jac1, and the monothiol glutaredoxin Grx5.

**FIGURE 5:**
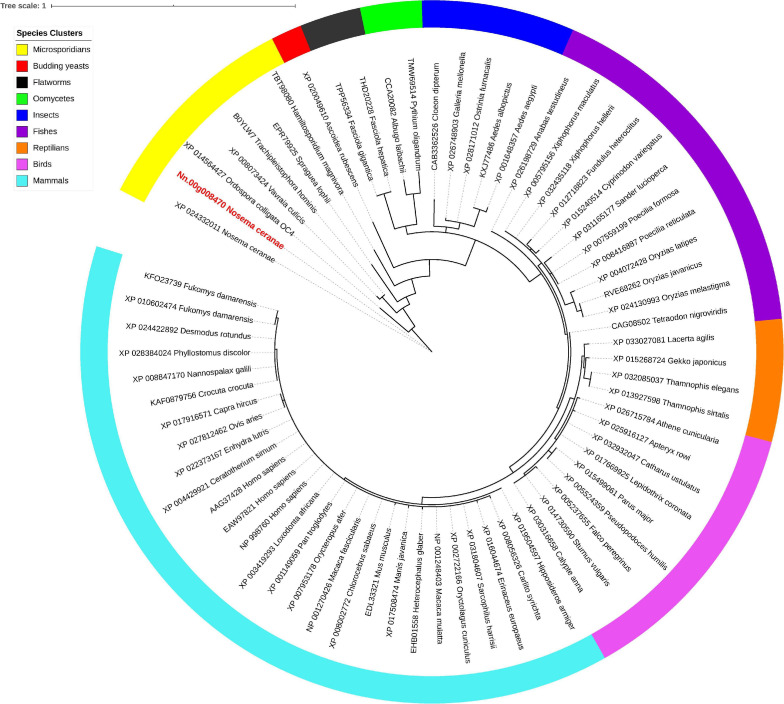
Maximum likelihood phylogenetic tree of Isu1 proteins. Branches are labeled as GenBank accession numbers followed by species names. Branches with bootstrap values (1,000 replicates) less than 0.5 were discarded. The genetic distance is drawn to scale. The current *Nosema ceranae* record is highlighted with red color. The definitions of the species clusters are adopted from the NCBI Taxonomy database.

**FIGURE 6:**
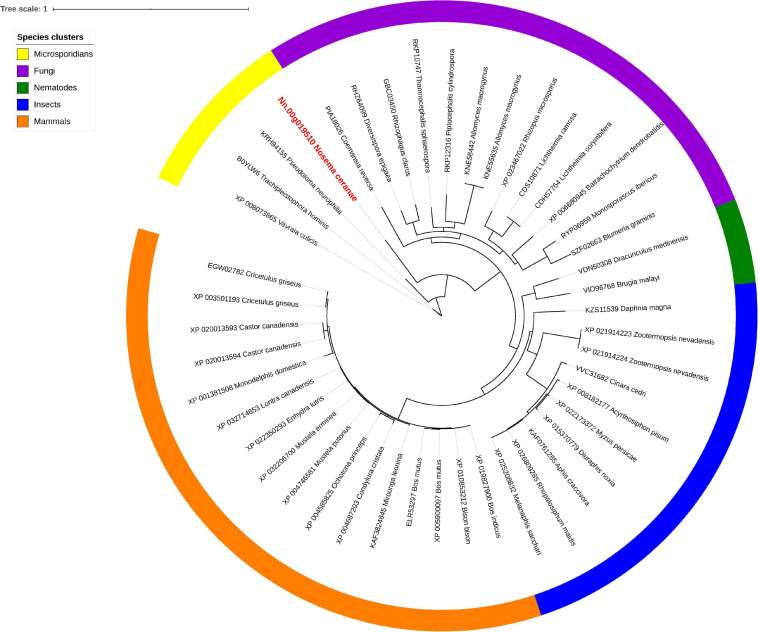
Maximum likelihood phylogenetic tree of Nfs1 proteins. Branches are labeled as GenBank accession numbers followed by species names. Branches with bootstrap values (1,000 replicates) less than 0.5 were discarded. The genetic distance is drawn to scale. The current *Nosema ceranae* record is highlighted with red color. The definitions of the species clusters are adopted from NCBI Taxonomy database.

**FIGURE 7:**
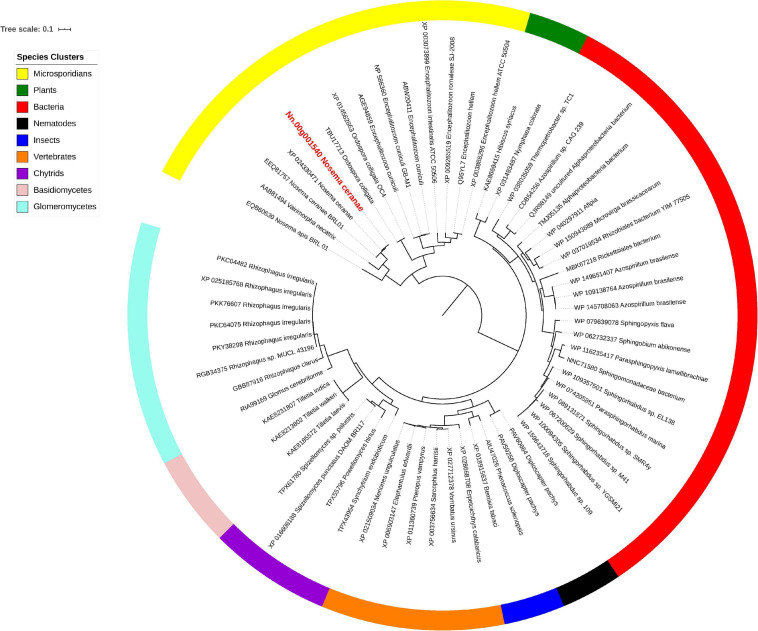
Maximum likelihood phylogenetic tree of Hsp70 proteins. Branches are labeled as GenBank accession numbers followed by species names. Branches with bootstrap values (1,000 replicates) less than 0.5 were discarded. The genetic distance is drawn to scale. The current *Nosema ceranae* record is highlighted with red color. The definitions of the species clusters are adopted from NCBI Taxonomy database.

Isu1, Nfs1, and Hsp70 are key components of Fe-S cluster assembly machinery. Orthologs for these proteins were retrieved to build the phylogeny trees crossing eukaryotic and prokaryotic organisms. By comparing the amino sequences, one significant hit was identified from the *N. ceranae* genome for Isu1 (Nn.00g008470, Figure 5), Nfs1 (Nn.00g019510, Figure 6), and Hsp70 (Nn.00g001540, Figure 7), respectively. The homologs of all three genes are inter-kingdom conserved, suggesting the conservation and importance of the Fe-S cluster assembly machinery. Generally, the genes from microsporidia (including *N. ceranae*) were relatively close and clustered. However, significant intra-microsporidian divergence was observed for Isu1 and Nfs1. Interestingly, all three phylogenetic trees suggest that the microsporidian sequences are branched relatively early from other species, indicating microsporidia may contain specific features of the Fe-S cluster assembly, supported by the Siddal and Whiting method (Supplementary Figure 1). A follow-up study will be to investigate the biosynthetic function of Fe-S proteins in the microsporidian mitosomes.

### Analysis of ATP-Binding Cassette (ABC) Transporter

ABC transporters constitute one of the largest superfamilies found in all living organisms, with the number of known members exceeding more than 10,000 species (Dassa and Bouige, 2001). ABC transporters contain a pair of nucleotide-binding domains (NBDs) that hydrolyze ATP and facilitate conformational alterations in the associated transmembrane domains (TMDs), thus permitting substrates to cross the membrane lipid bilayer and either be exported out of or imported into the cytoplasm (Locher, 2016). ABC transporter proteins are engaged in the ATP-dependent transport of extensive substrates across biological membranes, as well as receptors, ion channels, mRNA translation, and ribosome biogenesis (Kovalchuk and Driessen, 2010). Importantly, ABC transporters have been found to contribute to multidrug resistance in microbial pathogens and tumor cells (Piddock, 2006; Lubelski et al., 2007; Wu et al., 2019). According to the Human Genome Organization (HUGO) approved scheme, all eukaryotic ABC transporter proteins are categorized into eight major subfamilies (A to H) (Dean et al., 2001). In the fungal kingdom, the ABC transporter proteins have been well described in the budding yeast *S. cerevisiae* and the fission yeast *S. pombe* (Iwaki et al., 2006). However, knowledge of microsporidian ABC transporters remains limited. With the rapid development of genome sequencing projects in the past two decades, a diversity of ABC transporter proteins in genomes of microsporidian species has been uncovered, which allows for the comparative survey of ABC transporters within this group of organisms.

**FIGURE 8:**
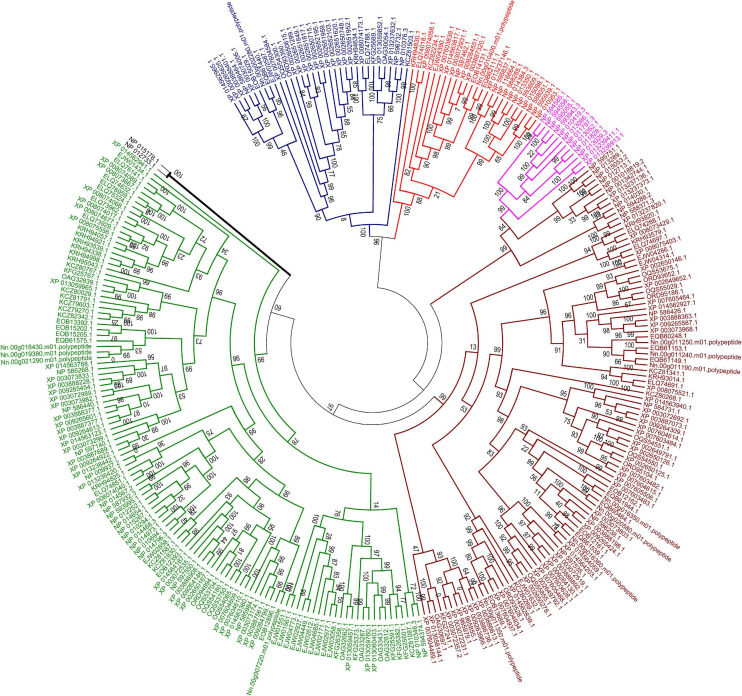
Maximum likelihood phylogenetic tree of ABC transporter proteins of 23 fungal genomes. The tree was based on a total of 293 ABC transporter protein sequences. The unrooted tree was calculated with FASTTREE based on JTT mode with 1,000 replications. The color code associated with each protein indicated the subfamily, with brown for subfamily B, purple for subfamily C, blue for subfamily E, red for subfamily F and green for subfamily G. Two ABC proteins from subfamily D in *S. cerevisiae* were labeled with black line.

In the current research, five of eight subfamilies (ABC-B, ABC-C, ABC-E, ABC-F, and ABC-G) were present in genomes of 21 microsporidian species, and among these ABC subfamilies, members of ABC-G proteins were the most abundant, followed by ABC-B subfamily (Table 2). ABC-A, ABC-D, ABC-H subfamilies seem to be lost in most or all microsporidia. ABC-C only presents in the basal microsporidian *M. daphniae*. ABC-B proteins represent a large category of ABC transporters, which are widely distributed among eukaryotes, including fungi. Their diverse functions are associated with the export of mitochondrial peptides, biogenesis of iron-sulfur (Fe-S) cluster proteins, multidrug resistance, and antigen processing (Kovalchuk and Driessen, 2010). Genomic investigations demonstrate that reduction in metabolic capabilities is the dominating feature of microsporidian genome evolution, which has been supported by expanding transporter gene families to compensate for pathway loss (Heinz et al., 2012; Nakjang et al., 2013; Freibert et al., 2017). In our study, ABC-B members were identified in all microsporidian genomes though the number varies among different species, suggestive of the importance of ABC-B family in microsporidia (Table 2 and Figure 8). The number of ABC-B members was conserved in *Encephalitozoon* species, suggesting an essential function in this group of microsporidia. However, the number of ABC-B has diverged within *Nosema* family, where *Nosema ceranae* maintained the highest number of ABC-B paralogs, indicative of a lineage-specific duplication of this subfamily, which can also be seen in the evolutionary tree (Figure 8). Alternatively, the lower number of ABC-B might be due to incompletely assembled genomes in other *Nosema* species. Considering that *N. ceranae* has become a globally predominant microsporidian species in honey bees, it’s of great interest to perform additional studies to explore the relationship between the high number of ABC-B and the high virulence of *N. ceranae*.

**TABLE 2:** Numbers of ABC transporter genes in 22 microsporidian species and two yeast species, in each of five subfamilies.

	Species	Subfamily					Total
		B	C	E	F	G	
Microsporida	*Encephalitozoon romaleae*	5	0	1	1	6	13
	*Encephalitozoon hellem*	6	0	1	1	6	14
	*Encephalitozoon intestinalis*	6	0	1	1	6	14
	*Encephalitozoon cuniculi*	6	0	1	1	5	13
	*Ordospora colligata*	5	0	1	1	5	12
	***Nosema ceranae***	**7**	**0**	**1**	**1**	**4**	**13**
	*Nosema apis*	6	0	1	1	1	9
	*Nosema bombycis*	3	0	1	1	5	10
	*Enterocytozoon bieneusi*	7	0	11	0	1	19
	*Enterospora canceri*	4	0	1	0	1	6
	*Enterocytozoon hepatopenae*	5	0	1	0	4	10
	*Vittaforma corneae*	12	0	1	1	2	16
	*Trachipleistophora hominis*	3	0	1	1	7	12
	*Vavraia culicis*	3	0	1	1	7	12
	*Pseudoloma neurophilia*	3	0	1	1	7	12
	*Edhazardia aedis*	3	0	1	1	12	17
	*Anncaliia algerae*	3	0	1	1	9	14
	*Nematocida* spERTm5	1	0	1	0	5	7
	*Nematocida* sp1	1	0	1	0	6	8
	*Nematocida parisii*	1	0	1	0	4	6
	*Mitosporidium daphniae*	3	2	1	1	2	9
Yeast	*Schizosaccharomyces pombe*	5	4	1	5	3	18
	*Saccharomyces cerevisiae*	4	6	1	5	11	29

*The bold means the assembled species in this study.*

ABC-G transporters in fungal species were often reported to be engaged in drug resistance and lipid translocation (Smriti et al., 2002; Coleman and Mylonakis, 2009). The number of ABC-G in the investigated microsporidian species was diverse, from only a single copy in *N. apis*, *E. bieneusi*, *E. canceri*, and *V. culicis* to 12 copies in *E. aedis* (Table 2). There might be some host- or environment-driven pressures to explain the huge birth and death rates of genes in this essential protein family, suggesting that this subfamily has undergone lineage-specific divergence during microsporidian evolution. *N. ceranae* (four ABC-G) showed higher copy number of ABC-G than that of its most closely related species *N. apis* (one ABC-G). However, the other three ABC-G orthologs were clustered into a single group (Figure 8), which indicates a recent gene duplication in ABC-G of *N. ceranae*. In another closely related species, *N. bombycis*, ABC-G was constantly expressed over the entire proliferation stages and was a key player in substrate transportation from ions to proteins (He et al., 2019). It is then interesting to decipher the detailed function of ABC-G during *N. ceranae* proliferation.

### Analysis of ATP/ADP Carriers

As the result of an intracellular lifestyle, the microsporidian parasites have lost canonical mitochondria and oxidative phosphorylation pathway; hence glycolysis is the mean to produce ATP (Timofeev et al., 2020). To satisfy their energy demands, microsporidia acquired the capability to import ATP directly from the host cell cytoplasm during proliferation (Tsaousis et al., 2008). Indeed, microsporidia have frequently been detected to be surrounded by host mitochondria (Han et al., 2019). ATP/ADP carriers, which were gained via HGT from intracellular bacteria, play a pivotal part in the transportation of ATP from infected cells to *E. cuniculi* (Tsaousis et al., 2008; Heinz et al., 2014). Additional efforts are required to conclude whether this is a common strategy during the evolution of microsporidian species.

**FIGURE 9:**
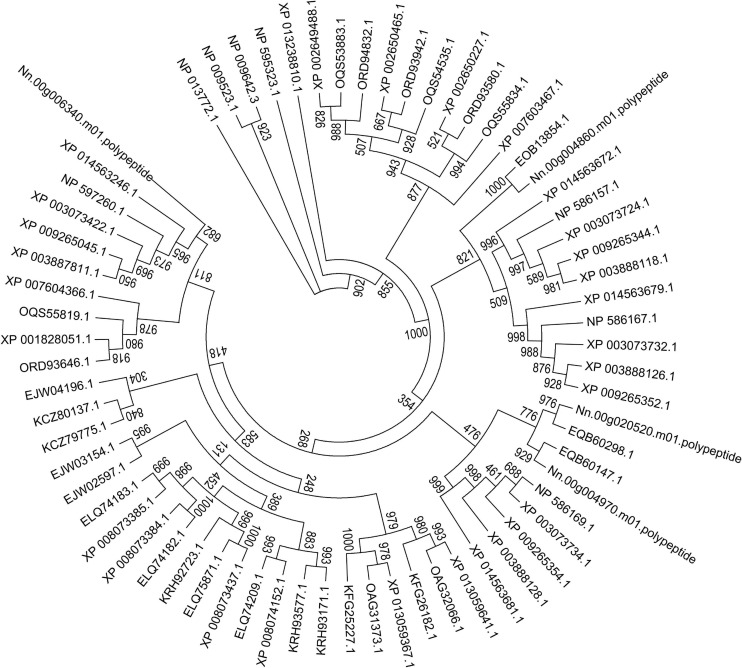
Maximum-likelihood phylogenetic tree generated based on ATP/ADP carrier proteins in 23 fungal genomes. Based on the total aligned 68 ATP/ADP carrier proteins, the tree, rooted with ATP/ADP carrier in *S. cerevisiae* and *S. pombe*, was calculated with PhyML based on JTT mode with 1,000 replications. Bootstrap values are indicated at the base of each clade.

Here, a phylogenetic tree of 21 microsporidian species and two yeast species *S. cerevisiae* and *S. pombe* was built based on the ATP/ADP carriers (Figure 9). There were three ATP/ADP carriers in *P. neurophilia* and *E. aedis*, similar to the number of ATP/ADP carriers identified in *S. cerevisiae* and *S. pombe*. Two members of the ATP/ADP carrier family were identified in *A. algerae*, *T. hominis*, *Nematocida* sp1, *Nematocida* spERTm5, and *N. parisii.* Only one ATP/ADP carrier was found in *M. daphnia*, which has a microsporidia-like morphology and is regarded as a basal microsporidia species (Bass et al., 2018). The different number of ATP/ADP carriers among various microsporidian parasites demonstrated that the selection of ATP/ADP carrier genes occurs during the lineage divergence. Species of the genus *Encephalitozoon* are ubiquitous vertebrate pathogens except for *E. romaleae*, which has been isolated from a grasshopper (Corradi, 2015). These species are well-known for their miniature genomes (ranging from 2.3 to 2.9 Mb) with the smallest coding capacity (Pombert et al., 2012). We observed that the numbers of ATP/ADP carrier members in *Encephalitozoon* species are highly conserved, and *E. cuniculi*, *E. bieneusi*, *E. hepatopenaei*, *E. intestinalis*, *E. hellem*, and *E. romaleae* all have four ATP/ADP carrier proteins. Similar to *Encephalitozoon* species, *N. ceranae* has four ATP/ADP carriers, which was higher than *N. apis* (two ATP/ADP carriers) and *N. bombycis* (one ATP/ADP carrier). *N. ceranae* Nn.00g020520 and Nn.00g004970 are, respectively, homologous to *N. apis* EQB60298.1 and EQB60147.1, while *N. ceranae* Nn.00g004860 is homologous to *N. bombycis* EOB13854.1 (Figure 9). Additionally, *N. ceranae* Nn.00g006340 has no homolog in *N. apis* or *N. bombycis*. This is suggestive of a recent lineage-specific gene expansion of ATP/ADP carrier family in *N. ceranae*. *N. ceranae* and *A. mellifera* have evolved together over just a short period, and arguably this imbalance explains why *N. ceranae* exerts more energetic stress on the bee host (Martin-Hernandez et al., 2011).

Due to a lack of well-established tools for genetic manipulation, functional study on ATP/ADP carriers in microsporidian is extremely limited, especially in *Nosema* species. Given the severe influence of *N. ceranae* on the worldwide beekeeping industry, it’s necessary and significant to carry out experimental work on the function of identified members of ATP/ADP carrier family in *N. ceranae* adopting molecular approach such as RNAi, which has been proved to be efficient in knockdown of several *N. ceranae* genes (Paldi et al., 2010; Li et al., 2016; Rodriguez-Garcia et al., 2018; Huang et al., 2019). We previously used purified spores of *N. bombycis* to infect *Bombyx mori* BmN cells, followed by transfection with a non-transposon vector pIZT/V5-His vector and the exogenous *gfp* gene was successfully inserted into the *N. bombycis* genome (Guo et al., 2016). Additional studies reported the successful establishment of the gypsy moth (*Porthetria dispar*) IPL-LD-65Y cell-based system of *N. ceranae* infection (Gisder et al., 2011; Gisder and Genersch, 2015), which provides an excellent opportunity to conduct functional exploration of ATP/ADP carrier genes of *N. ceranae*.

### Synteny Analysis of the Genes Coding Spore Wall Proteins (SWP) and Polar Tube Proteins (PTP)

**FIGURE 10:**
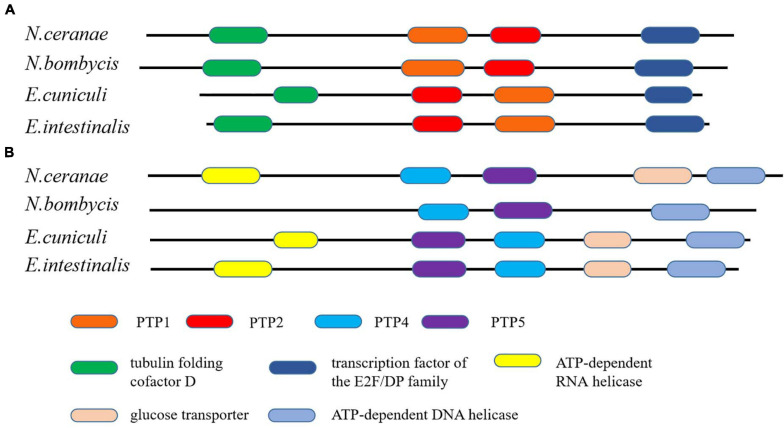
The conservation of gene order among *N. ceranae* and three other related microsporidian species. **(A)** The distribution of PTP1 and PTP2 genes in the synteny block. **(B)** The distribution of PTP4 and PTP5 genes in the synteny block. The colored oblong represents genes with functional annotations.

Microsporidia infects host cells by employing a unique, highly specialized invasion device including the spore wall (SW) and polar tube. After germination, the polar tube protein PTP1 can interact with lectin receptors on the host cell surface (Xu et al., 2003, 2004). PTP2 with a basic lysine-rich core was clustered closely with PTP1 on a contig. PTP3 was up-regulated during sporogony at the transcriptional level (Peuvel et al., 2002). Polar tube protein 4 (PTP4) has been demonstrated to have a specific epitope on the tip of the PT, and this epitope was shown to interact with the host cell transferrin receptor (TfR1) (Han et al., 2017). The five known genes encoding the polar tube protein were all present in the current genome assembly, which include PTP1, PTP2, PTP3, PTP4, PTP5 genes. After determining the homologous gene loci in the genome, one syntenic block harboring the PTP1 and PTP2 was identified between *N. bombycis*, *N. ceranae*, *E. cuniculi* and *E. intestinalis* (Figure 10). Remarkably, the PTP1 and PTP2 were arrayed conversely both in *N. ceranae* and *N. bombycis* compared with *E. cuniculi* and *E. intestinalis* (Supplementary Figure 2). Similarly, another syntenic block, including the PTP4 and PTP5 genes, was identified (Figure 10). According to the composition of the spore wall, there are multiple SWPs in both the exospore and endospore (Han et al., 2020). In the current assembly, seven genes encoding the spore wall protein were identified based on homologous searches with BLASTP (Table 3). Protein domain prediction showed that NcSWP12 contains BAR/IMD domain which served as sensors of membrane curvature (Quinones et al., 2010). In *N. bombycis*, NbSHWP12 with BAR/IMD domain is localized to the spore wall and can adhere to the deproteinized chitin coats (Chen J. et al., 2013). The homologous gene to NcHSWP7, NbSWP7, is localized in the exospore, endospore and polar tube of the mature *N. bombycis* spores where it mediates adherence to host cells (Yang et al., 2015). NcHSWP1, which is the ortholog of EnP1, was found to contain the heparin-binding motif (HBM), which mediates interactions between spores and glycosaminoglycan from the surface of host cells (Southern et al., 2007). The low number of conserved spore wall genes might be due to high divergence of these components among microsporidian species.

**TABLE 3:** The identified spore wall proteins and Polar tube proteins of *Nosema ceranae*.

Protein	Subcellular locationa	Function domain	Mw (kDa)	Amino acids (aa)	pI	GenBank ID
HSWP1	Endospore	mobidb-lite HBM, PF14239, RRXRR Domain of unknown function (DUF4770)	48.42	426	8.49	G9061_00g010360
HSWP2		Pentapeptide repeats	30.97	265	9.3	G9061_00g004470
HSWP3		Signal-pep	41	341	8.5	G9061_00g006590
HSWP4		Transmembrane	52.8	475	6.84	G9061_00g007610
HSWP7	Exospore, endospore and polar tube	SCOP domain d1ktba1	31.8	281	4.56	G9061_00g011170
HSWP9	Exospore, endospore and polar tube	Transmembrane helix region (TMHMM)	44.37	379	9.74	G9061_00g017340
HSWP12	Exospore and endospore	BAR/IMD domain	26.7	228	8.17	G9061_00g008430
spore wall protein precursor		Signal-pep	25.79	222	5.14	G9061_00g009010
NcPTP1		Methylene-tetrahydrofolate reductase C terminal	46.87094	456	4.92	G9061_00g021140
NcPTP2			30.44299	275	9.51	G9061_00g021150
NcPTP3			157.55451	1,414	6.46	
NcPTP4			24.02931	208	5.86	G9061_00g019220
NcPTP5			31.78817	268	9.05	G9061_00g019230

*N. ceranae* destroys the gut integrity of honey bees, leading to impaired flying and memory abilities, which can lead to the loss of colonies. We anticipate that the updated genome resource and comparative analyses provided here lead to novel methods to control this parasite without harming honey bees. The provided genome also reveals numerous evolutionary features compared with other microsporidian parasites, which may help to clarify the evolution of virulence and co-evolution with hosts.

## Data Availability Statement

The datasets presented in this study can be found in online repositories. The names of the repository/repositories and accession number(s) can be found below: https://www.ncbi.nlm.nih.gov/, SRR8536193.

## Ethics Statement

The apiaries for bee sample collection are the property of the USDA-ARS Bee Research Laboratory, Beltsville, MD, United States. No specific permits are required for the described studies. Studies involved the European honey bee (*Apis mellifera*), which is neither an endangered nor a protected species.

## Author Contributions

QH and JE designed the investigation. QH assembled the genome. WL performed Fe-S phylogenetic analysis. RG performed ABC and ATP/ADP carriers’ phylogenetic analysis. JX, XD, and ZM performed SW and Polar tube protein phylogenetic analysis. QH, ZW, WL, RG, JX, XD, ZM, YC, and JE wrote the manuscript. All authors contributed to the article and approved the submitted version.

## Conflict of Interest

The authors declare that the research was conducted in the absence of any commercial or financial relationships that could be construed as a potential conflict of interest.

## Footnotes

1 https://www.ncbi.nlm.nih.gov/nuccore/AOMW00000000.2

2 https://www.ncbi.nlm.nih.gov/nuccore/AEYK00000000.1

3 http://tree.bio.ed.ac.uk/software/figtree/

## Supplementary Material

Supplementary File 1 Additional results.

Supplementary File 2 *De novo* transcriptome assembly.

Supplementary File 3 Genes in SVs.

Supplementary File 4 Sequences for microsporidian phylogenetic tree of Figure 2.

Supplementary File 5 Sequences for *Dicer* phylogenetic tree of Figure 4A.

Supplementary File 6 Sequences for Dicer up and down stream phylogenetic tree of Figure 4B.

Supplementary File 7 Sequences for ISU1 phylogenetic tree of Figure 5.

Supplementary File 8 Sequences for NFS1 phylogenetic tree of Figure 6.

Supplementary File 9 Sequences for HSP1 phylogenetic tree of Figure 7.

Supplementary File 10 Sequences for ABC phylogenetic tree of Figure 8.

Supplementary File 11 Sequences for ATP phylogenetic tree of Figure 9.

Supplementary File 12 Sequences for PTP synteny of Figure 10.
