# Supplementary material for: Genome and Evolutionary Analysis of Nosema ceranae: A Microsporidian Parasite of Honey Bees
Source: Front Microbiol. 2021 Jun 2;12:645353. doi: 10.3389/fmicb.2021.645353 (PMC8206274; doi:10.3389/fmicb.2021.645353)
Supplement: Supplementary File 1 — Additional results. [file Data_Sheet_1.ZIP › S1 supplementary results.docx]

Genome and evolutionary analysis of *Nosema ceranae*: a microsporidian parasite of honey bees

Qiang Huang^*#1,2^, Zhi Hao Wu^#1,2^, Wen Feng Li^3^, Rui Guo^4^, Jin Shan Xu^5^, Xiao Qun Dang^5^, Zheng Gang Ma^5^, Yan Ping Chen^6^, Jay D. Evans^6^

^1^ Honeybee Research Institute, Jiangxi Agricultural University, Zhimin Ave. 1101, Nanchang, 330045, China.

^2^ Jiangxi Province Key laboratory of Honeybee Biology and Beekeeping, Jiangxi Agricultural University, Zhimin Ave. 1101, Nanchang, 330045, China.

^3^ Guangdong Key Laboratory of Animal Conservation and Resource Utilization, Guangdong Public Laboratory of Wild Animal Conservation and Utilization, Guangdong Institute of Applied Biological Resources, Guangzhou 510260, China

^4^ College of Animal Sciences (College of Bee Science), Fujian Agriculture and Forestry University, Shangxiadian Road 15, Fuzhou 350002, China.

^5^ College of Life Science, Chongqing Normal University, Chongqing 401331, China.

^6^ USDA-ARS Bee Research Laboratory, BARC-East Building 306, Beltsville, Maryland, 20705, USA.

*for correspondence: [qiang-huang@live.com](mailto:qiang-huang@live.com) (Q.H.)

#the authors contributed equally

Results


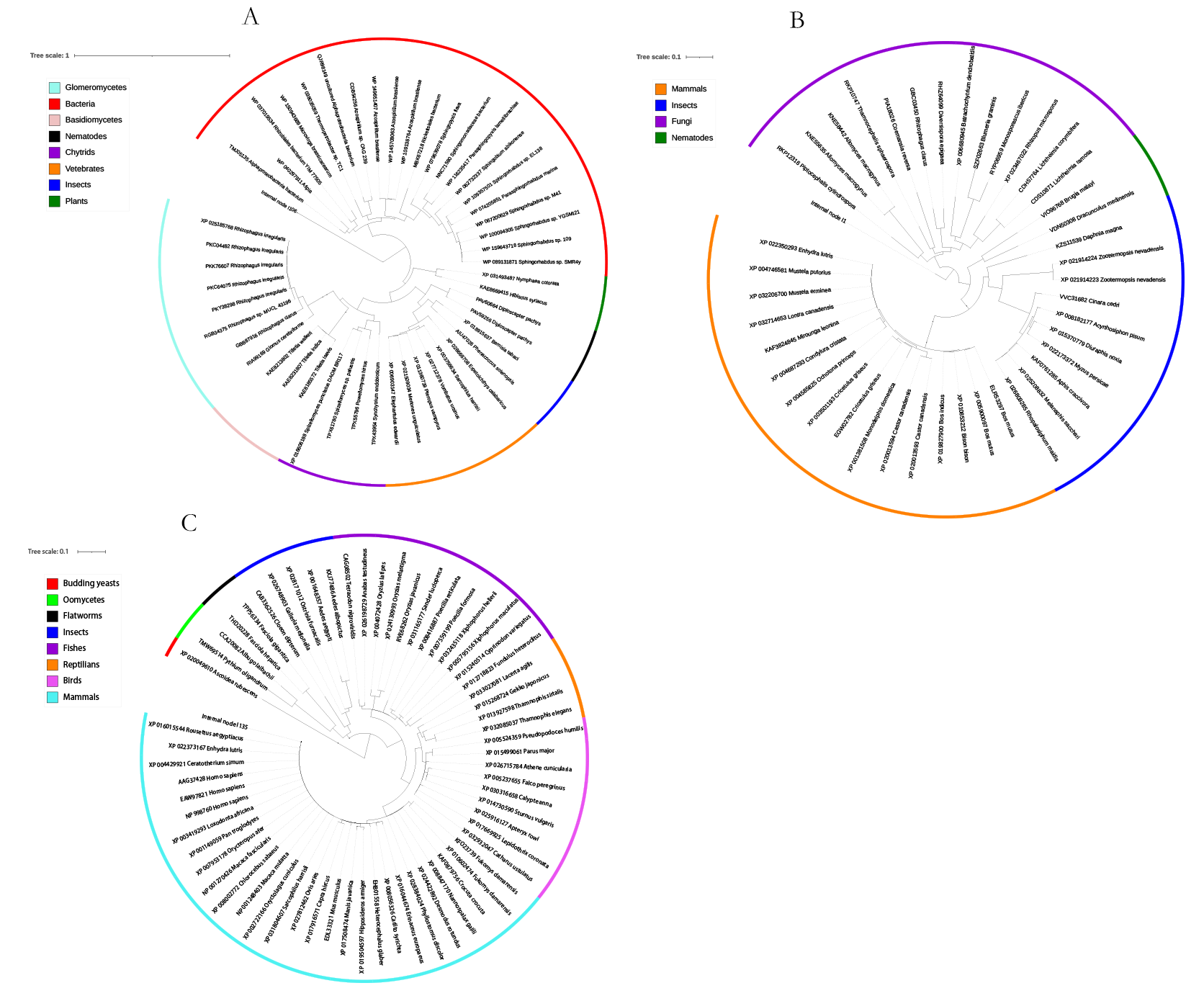


Figure S1 Maximum likelihood phylogenetic tree of HSP70 proteins (A), NFS1 protein (B), and Isu1 proteins (C). The microsporidian species have been removed and the topology remains congruent, which could exclude the long branch attraction effect.


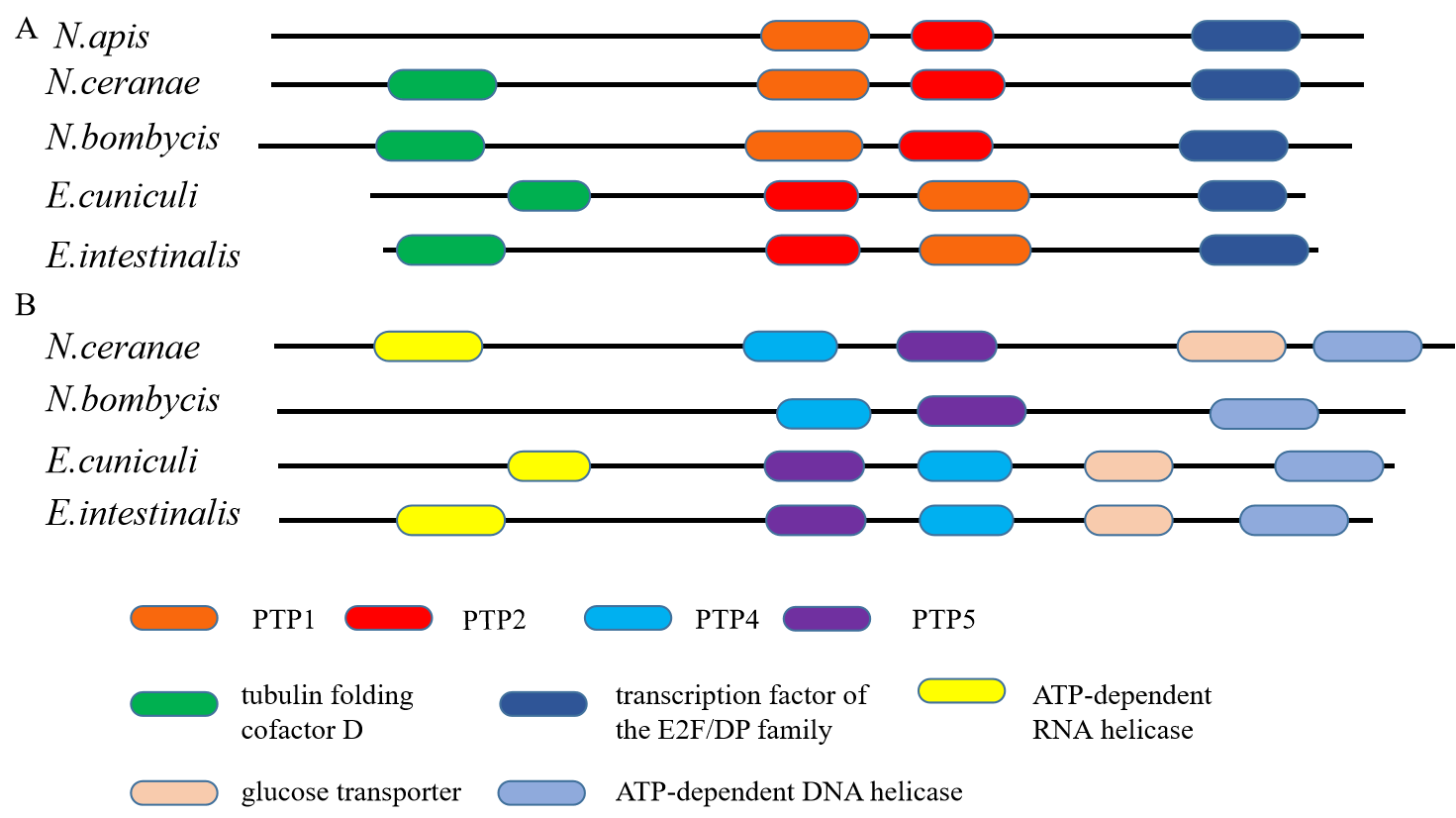


Figure S2 The conservation of gene order among *N. ceranae* and four other related microsporidian species. A: the distribution of PTP1 and PTP2 genes in the synteny block. B: The distribution of PTP4 and PTP5 genes in the synteny block. The colored oblong represents genes with functional annotations.

Table S1 Genome statistic for the studied microsporidian species with annotation. There are few recently sequenced microsporidia genomes.

|  | Genome size [Mbp] | Number of protein coding genes | Number of Intron | Intergenic size [Mbp] | Releasing year |
| --- | --- | --- | --- | --- | --- |
| *Nosema ceranae* | 8.8 | 2280 | 0 | 6.4 | 2019 |
| *Edhazardia aedis* | 51.3 | 4212 | 15 | 46.2 | 2015 |
| *Nosema bombycis* | 15.6 | 4468 | 2 | 12.2 | 2013 |
| *Anncaliia algerae* | 12.1 | 3598 | 1 | 9.1 | 2014 |
| *Nosema apis* | 8.5 | 2764 | 0 | 5.7 | 2013 |
| *Trachipleistophora hominis* | 8.4 | 3212 | 0 | 5.5 | 2013 |
| *Vavraia culicis* | 6.1 | 2777 | 4 | 2.8 | 2011 |
| *Spraguea lophii* | 5.7 | 2499 | 8 | 3.1 | 2013 |
| *Mitosporidium daphniae* | 5.6 | 3287 | 0 | 1.4 | 2014 |
| *Pseudoloma neurophilia* | 5.2 | 3644 | 1 | 2.3 | 2015 |
| *Nematocida parisii* | 4 | 2726 | 0 | 0.7 | 2012 |
| *Enterocytozoon bieneusi* | 3.8 | 3632 | 0 | 1.1 | 2009 |
| *Enterocytozoon hepatopenaei* | 3.2 | 2536 | 0 | 0.8 | 2017 |
| *Vittaforma corneae* | 3.2 | 2241 | 0 | 1.1 | 2011 |
| *Enterospora canceri* | 3 | 2169 | 1 | 1.2 | 2017 |
| *Encephalitozoon intestinalis* | 2.2 | 1968 | 1 | 0.2 | 2010 |
| *Encephalitozoon cuniculi* | 2.2 | 1870 | 1 | 0.2 | 2015 |
| *Encephalitozoon hellem* | 2.2 | 1882 | 1 | 0.1 | 2012 |
| *Encephalitozoon romaleae* | 2.1 | 1864 | 1 | 0.1 | 2012 |
| *Ordospora colligata* | 2.2 | 1879 | 1 | 0.3 | 2014 |

Table S2 Mitosomal orthologs in microsporidian species

| Microsporidian species | Isu1 | Nfs1 | Isd11 | Yfh1(frataxin) | Yah1(ferredoxin) | Ssq1(Hsp70) | Grx5 |
| --- | --- | --- | --- | --- | --- | --- | --- |
| *Nosema ceranae* | G9061_00g008470 | G9061_00g019510 | G9061_00g013760 |  | G9061_00g007050 | G9061_00g001540G9061_00g014940 G9061_00g018730 | Nn.00g006380 |
| *Nosema apis* | EQB61902 | EQB60279 |  | EQB60682 |  | EQB60639, EQB60092, EQB59878 | EQB60303 |
| *Nosema bombycis* | EOB12699, EOB14424 | AET97827 |  | EOB13743, EOB11450 | EOB14724, EOB14732 | AGQ46525, BAF76326, AEO23040 |  |
| *Antonospora locustae* | AAY27407 | AAY27413 |  | AAY27415 |  | AAC47660, AAY27406, AAT12379 |  |
| *Trachipleistophora hominis* | B0YLW7 | B0YLW6 | B2C6F2, ELQ76067 | B0YLW8 | ELQ76652 | AAM97590, ELQ75191, ELQ76668 | ELQ76118 |
| *Vittaforma corneae* | XP_007604698, XP_007604136 | XP_007604381, XP_007604382 |  |  | XP_007603620 | XP_007603681, XP_007603680, XP_007605309, XP_007604183, XP_007605310 | XP_007604320 |
| *Encephalitozoon cuniculi* | XP_965885 | NP_586483 | KMV65350 | XP_965969 | NP_585988 | ABW20411, AGE94859 |  |
| *Encephalitozoon intestinalis* | XP_003072261 | XP_003074026 | XP_003073659 | XP_003072346 | XP_003073178 | XP_003073899, XP_003072547, XP_003072363 |  |
| *Edhazardia aedis* | EJW01541 | EJW02249 |  | EJW03997 | EJW02180 | EJW05229, EJW05228, EJW04430, EJW02229, EJW04429 | EJW03582 |
